# Analysis of the sample size used in clinical MRI studies

**DOI:** 10.1371/journal.pone.0316611

**Published:** 2025-03-03

**Authors:** Clara M Bögerl, Frederik B Laun, Armin M Nagel, Sebastian Bickelhaupt, Michael Uder, Jannis Hanspach

**Affiliations:** Institute of Radiology, Universitätsklinikum Erlangen, Friedrich-Alexander-Universität Erlangen-Nürnberg (FAU), Erlangen, Germany; Memorial Sloan Kettering Cancer Center, UNITED STATES OF AMERICA

## Abstract

**Background:**

Choosing the sample size in clinical MRI studies is a common, important, and challenging task, complicated by the substantial variation in potential study parameters. However, considering previously used sample sizes may provide a reference point for future studies. The purpose of the study was to systematically investigate and to provide orientation for sample size selection based on information from current practices in clinical MRI studies.

**Methods:**

We assessed 1,046 research articles published in the Journal of Magnetic Resonance Imaging (JMRI) between 2020 and 2023. Only studies that involved patients were included. Review articles and studies using phantoms, animals, ex vivo samples, publicly available datasets, and non-imaging techniques (e.g., spectroscopy) were excluded. The included studies were categorized according to various criteria including anatomical region, field strength, contrast category (e.g., T_1_ mapping or diffusion-weighted imaging), retrospective vs. prospective and single vs. multicenter studies, automatic or manual segmentation, and quantitative or qualitative evaluations.

**Results:**

The median sample size (=number of patients) of the 734 studies included in the analysis was 74.5 (retrospective studies =  129, prospective studies =  41) and varied between the investigated categories. Sample size clusters were found in multiples of ten (e.g., 20, 30, 40), and 90.3% of the studies had less than 350 patients with 50.5% having less than 75, while 1.6% had more than 1,000 patients.

**Conclusion:**

There is wide variation in the sample sizes of studies published by JMRI between 2020 and 2023, depending on study type, content category, or evaluation method. In clinical MRI studies, balancing statistical power and minimizing patient involvement is crucial, necessitating carefully choosing the sample size.

## Introduction

Van Beek et al. once said there is little doubt that magnetic resonance imaging (MRI) is one of the most powerful diagnostic tools in contemporary clinical medicine [1]. However, in the age of evidence-based medicine [2], the MRI research community must provide the necessary evidence to support this notion. The success and impact of various radiological journals, including the Journal of Magnetic Resonance Imaging (JMRI), suggests that this evidence is being reliably provided and using established procedures.

Nonetheless, the planning of clinical MRI studies faces several challenges, the most notable being sample size planning—which must be performed before the study begins. This pre-study planning must be defended before ethics committees and, typically, in front of funding agencies. Hence, it is pivotal to the success of the study. A sample size that is too small may not reach statistical certainty. Too large a sample size should also be avoided, especially for ethical reasons as MRI studies place the burden of increased scan time on patients who are often suffering from severe diseases. Moreover, spending more resources than necessary is environmentally and economically problematic [3] and may drain the working hours of medical professionals. This can be particularly challenging as “Healthcare workforce shortages are some of the biggest and most pressing challenges” confronting healthcare systems in many countries [4].

There are mathematical tools to estimate sample sizes. However, they require using prior knowledge such as expected measurement variability and effect sizes [5, 6] that may not always be available, especially when evaluating novel MRI techniques or when applying established MRI techniques to unexplored medical questions.

In a previous research article published in Magnetic Resonance in Medicine (MRM) in 2020, our group investigated the sample sizes (that is the number of healthy volunteers) used in technical development studies in the field of MRI. We found a median sample size of six, with sizes between one and ten the most commonly used [7]. However, this finding cannot be translated to clinical studies. Technical MRI studies often aim to demonstrate the technical feasibility of a new method or measurement setup, which can typically be shown with low sample sizes.

Therefore, this study aimed to provide a reference point for planning future clinical MRI studies by investigating the sample sizes used in previously published articles in one of the leading journals in the field, JMRI. Given that sample sizes in medical MRI studies depend on various cofactors, we also evaluated its dependencies on multiple variables including anatomical region, field strength, contrast category (e.g., T_1_ mapping or diffusion-weighted imaging (DWI)), retrospective vs. prospective study types, single vs. multicenter studies, the use of automatic or manual segmentation approaches, and quantitative and qualitative evaluations.

## Materials and methods

### Study selection

**Fig 1:**
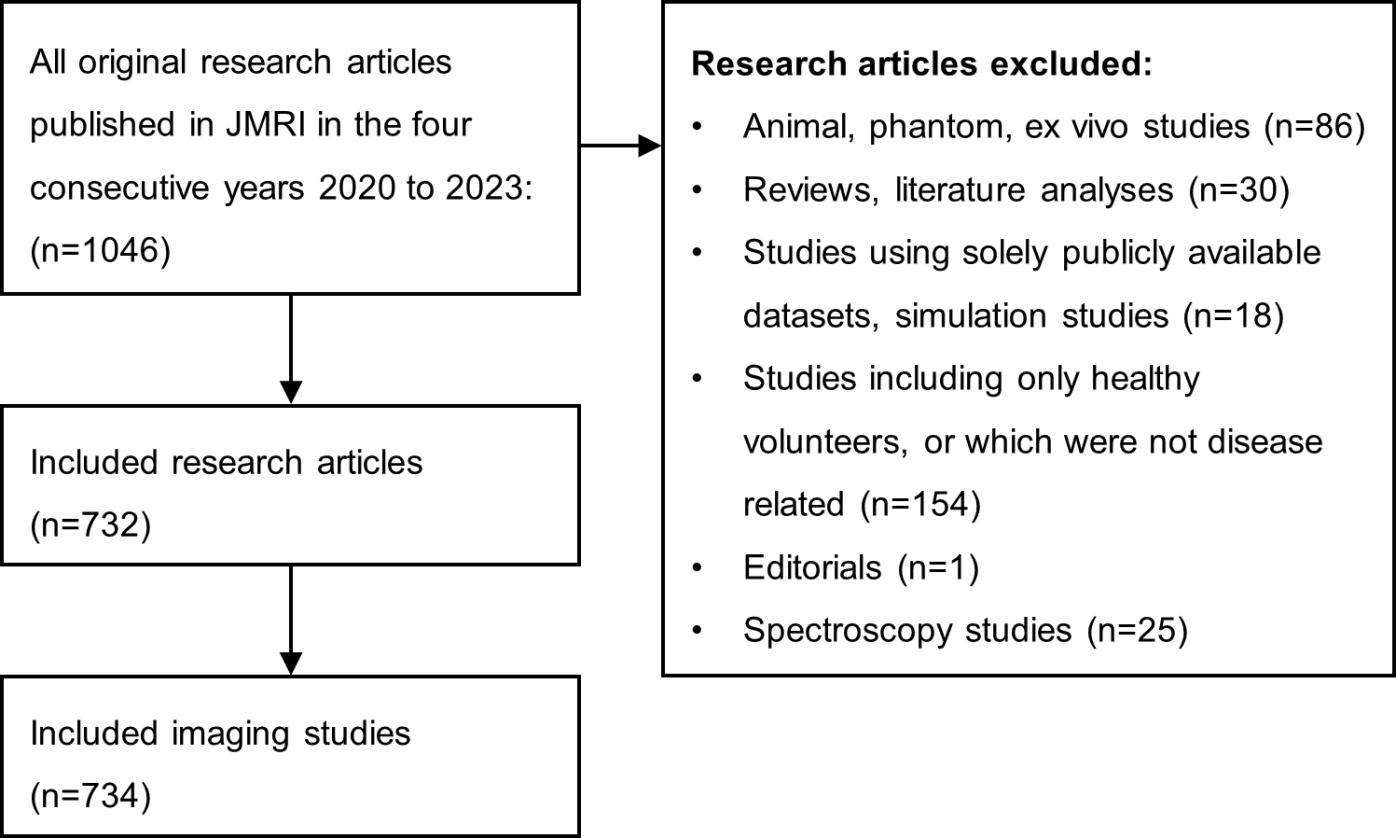
Study selection flowchart.

All research articles published in JMRI in the four consecutive years between 2020 and 2023 were collected and investigated (Fig 1). Of the 1,046 published research articles, only MR imaging studies that included patients were included for analysis. Here, ‘patients’ refers to individuals with a diagnosed illness or health problems related to the study’s research objective. We excluded systematic reviews, studies that used non-imaging techniques (e.g., spectroscopy), and studies that measured only animals, phantoms, or ex vivo tissue samples. We further excluded articles that used solely publicly available datasets. If a study investigated phantoms, healthy volunteers and/or patients, only the part investigating patients was included. Studies that treated two groups of patients differently were divided into separate studies. As a result, two additional studies were added [8, 9] to the 1046 published articles, which resulted in 1048 included studies.

No ethics approval was required for this study, as it did not include any experimental procedures involving humans or animals.

### Data extraction

Each study was categorized according to the sample size (number of patients), field strength, content category (i.e., anatomical region, given by *JMRI*), and MRI contrast. Twenty-five different MRI contrasts were defined for this purpose (S2 Table a,b). Additional information, specifically the study type (retrospective or prospective) was also extracted. Evaluation techniques were specifically defined (see Table 1) and extracted. Studies were further categorized based on whether they were conducted in multiple or single centers, and whether they delivered quantitative (techniques with quantitative measures: e.g., flow rate, relaxation times or spatial measurements) and/or qualitative results (e.g., categorization, qualitative rating).

**Table 1:** Number of studies and sample size per evaluation method.

Evaluation method	Number of studies	Percentage of studies	Sample size			
			Median	Mean	Min	Max
Qualitative rating	99	13.5%	50	107.3	1	1157
Scoring system	63	8.5%	75	166	3	1073
Machine learning	85	11.6%	226	427.1	3	5224
Radiomics	109	14.8%	180	231.8	5	941
Categorization	192	26.2%	95.5	232.5	3	6229
Spatial measurements	37	5.0%	125	172.2	19	575
Automatic segmentation	104	14.1%	55.5	120	8	1628
Manual segmentation	488	66.5%	79	170.1	1	6229
Semiautomatic segmentation	90	12.2%	57.5	93	3	547

Number of studies (and percentage of all studies) per evaluation method. Median, mean, minimum, and maximum value of the sample size per evaluation method.

### Statistical analysis

For each category, we calculated the number of studies and the mean, median, minimum, and maximum values of the sample sizes. The analyses of the contrast and content categories and the magnetic field strengths were further divided into prospective or retrospective studies. The relationship between the number of studies and the sample size was specifically analyzed and visualized to compare prospective and retrospective studies, multi- and single-center studies, those using manual or automatic segmentations, and studies relying on quantitative or qualitative data. The data were visualized using histograms and boxplots.

## Results

**Fig 2:**
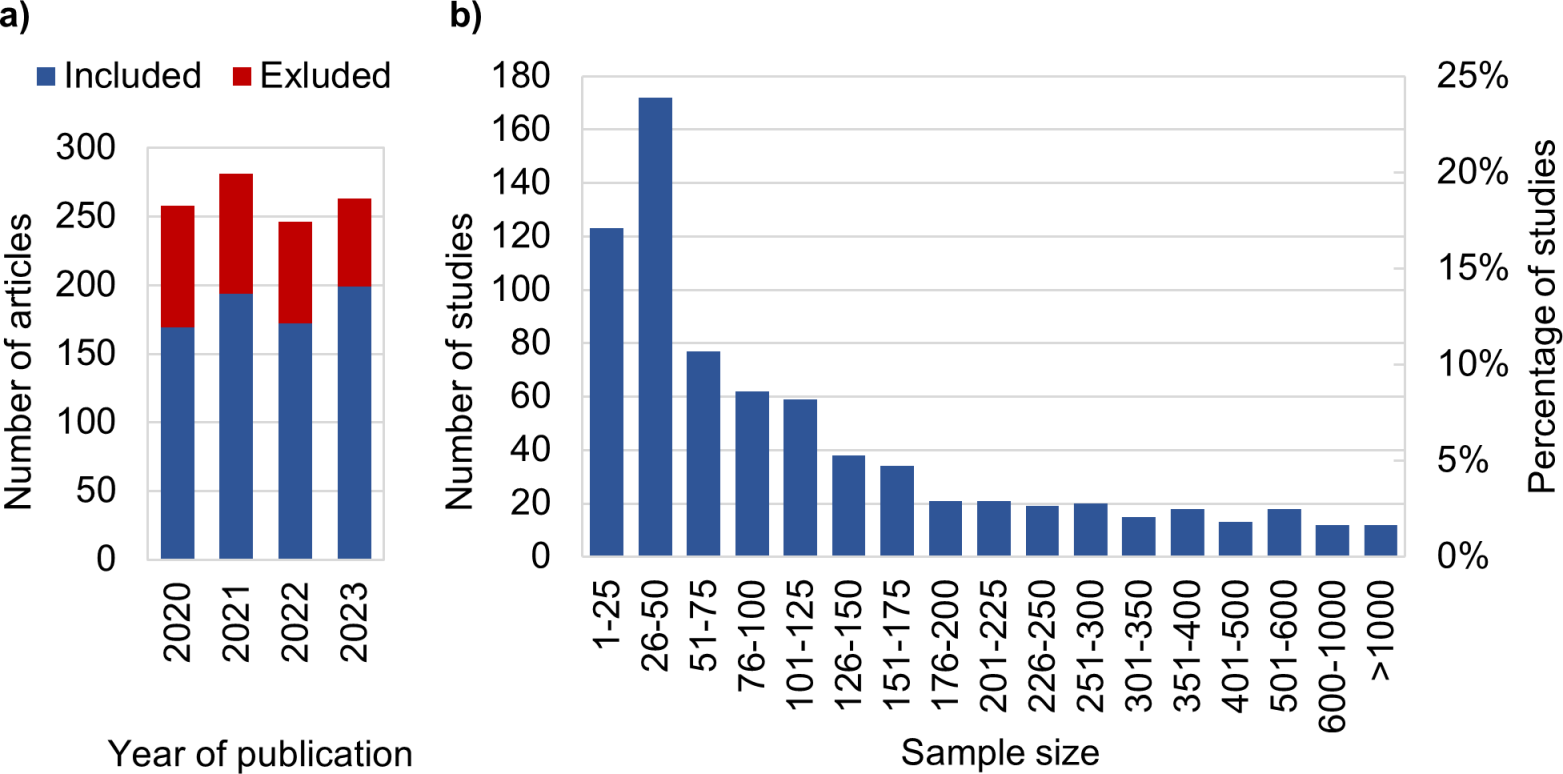
Number of articles and studies. Histogram of the number of articles published in JMRI from 2020 to 2023 and included (blue) and excluded (red) in this study (a). Histogram of the number of studies vs. sample size (b). To improve the visualization, the sample sizes were grouped on the x-axis. Numbers from 1 to 250 in intervals of 25, from 251 to 400 in intervals of 50, from 401 to 600 in intervals of 100, and from 601 to the maximum number in two intervals.

Of the 1048 published studies, 734 were included for analysis, and 314 were excluded based on the criteria described in Fig 1. S1 Table provides the collected data, together with a Excel-based tool to extract specific parts of the data (e.g., sample sizes of breast DWI). A histogram showing the number of published research articles each year is depicted in Fig 2a. Two hundred sixty-three articles were published in JMRI in 2023, 246 in 2022, 281 in 2021, and 258 in 2020. Fig 2b displays the aggregated number of articles from all years, categorized according to groups with similar sample sizes. S3 Table provides the respective numerical data. Of the included studies, 371 (50.5%) and 663 (90.3%) had sample sizes of less than 75 patients and 350 patients, respectively. Twelve studies (1.6%) included more than 1000 patients. Considering all 734 studies, the median sample size was 74.5 (mean =  157.8), the maximum was 6229 [10], and the minimum was 1 [11]. The median sample size in prospective studies was 41 (mean =  68.3, with 90% of the studies using a sample size < 151); and the median in retrospective studies was 129 (mean =  228.8). In 90% of the studies, the sample size was smaller than 490. In 245 studies (33.4%), both healthy volunteers and patients were examined.

**Fig 3:**
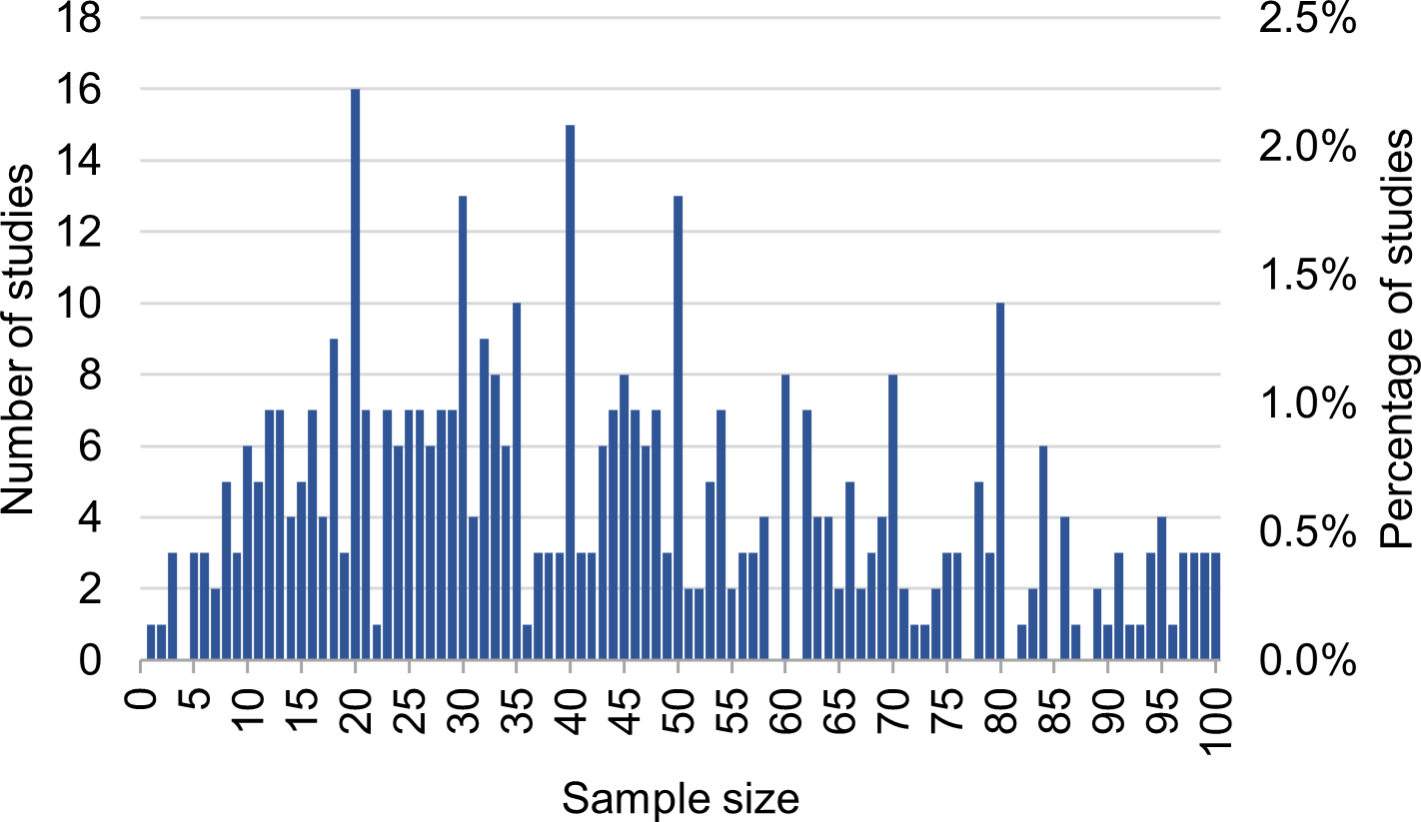
Histogram of the number of studies vs. the sample size. To improve the visualization, sample sizes > 100 were excluded.

Fig 3 shows a histogram of the same data as in Fig 2b but with bin sizes of 1 and a sample size smaller than 100 to allow for a more detailed look at the 59.1% of studies that used smaller sample sizes. The sample sizes approximating multiples of 10 (e.g., 20, 30, 40, etc.) had a higher occurrence (see peaks in Fig 3). The most frequent sample size was 20, which occurred in 16 studies (2.2%), followed by 40 in 15 studies (2.0%).

### Content category

**Fig 4:**
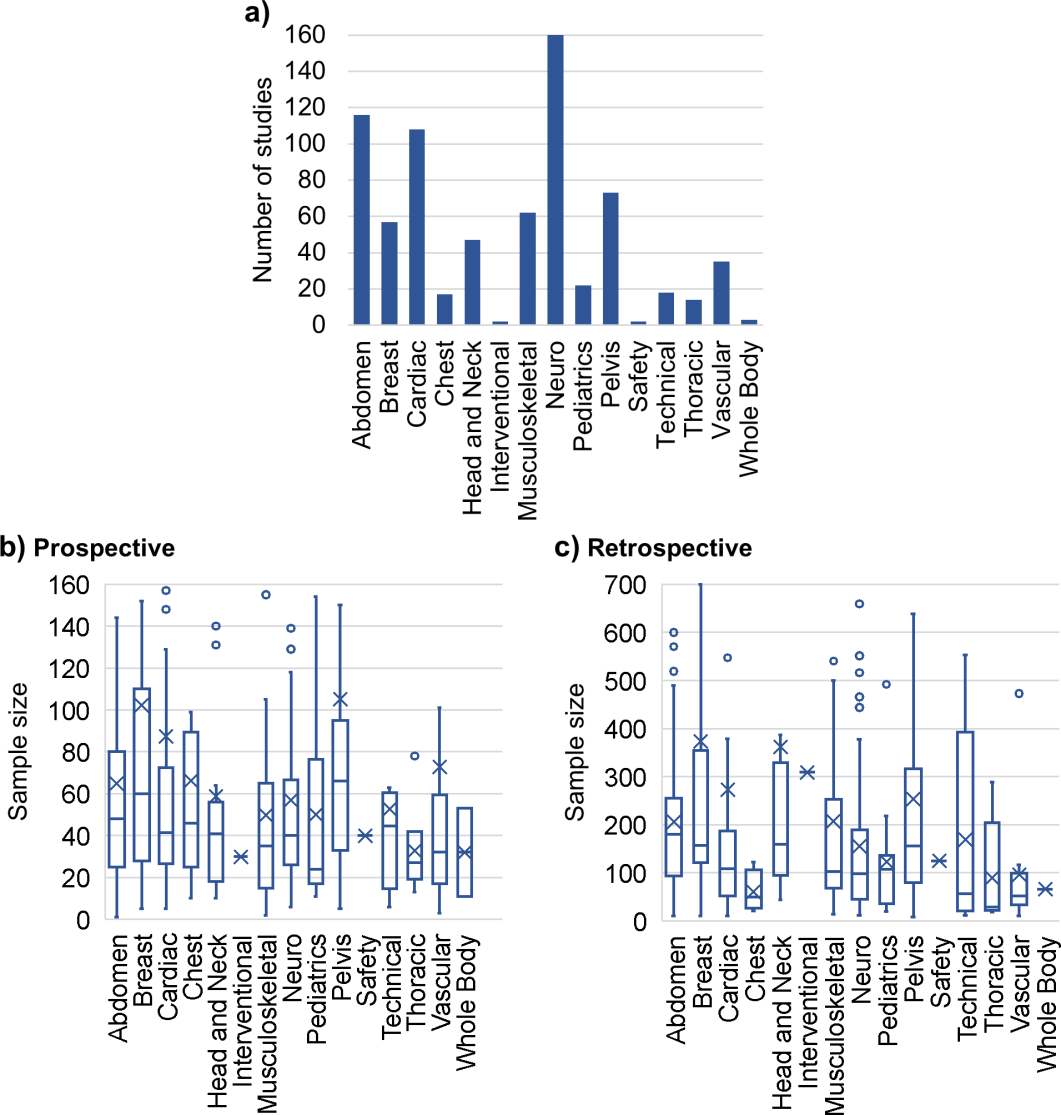
Content category. Histogram of the number of studies in each content category (a) and boxplots (displaying the minimum, maximum, first quartile, median, third quartile, mean, and outliers) of the sample sizes in prospective (b) and retrospective (c) studies per content category. Outliers are partially not visible.

Fig 4a shows the number of studies per content category and S4 Table provides the respective numerical data. The highest number of studies was found in the *Neuro* category with 161 studies, followed by *Abdomen* (115 studies) and *Cardiac* (107 studies). Fig 4b,c show the sample sizes in prospective and retrospective studies, respectively. The categories *Interventional* and *Safety* contain one retrospective and one prospective study each, and the category *Whole Body* contains one retrospective and two prospective studies. The prospective categories with the largest median sample sizes were *Breast* (median = 60, mean = 102.3) and *Pelvis* (median = 66, mean = 105.2). In contrast, *Pediatrics* had the lowest median sample size among the prospective studies, with a median of 24 (mean = 50.1).

The highest median sample size in retrospective studies (Fig 4c) was found in the *Abdomen* category (median = 180, mean = 206.1), followed by *Head and Neck* (median = 159.5, mean = 362.2), except for *Interventional* (N = 1) [12]. The lowest median sample size among the retrospective studies was in the *Thoracic* category (median = 29, mean = 89.4).

### Field strength

**Fig 5:**
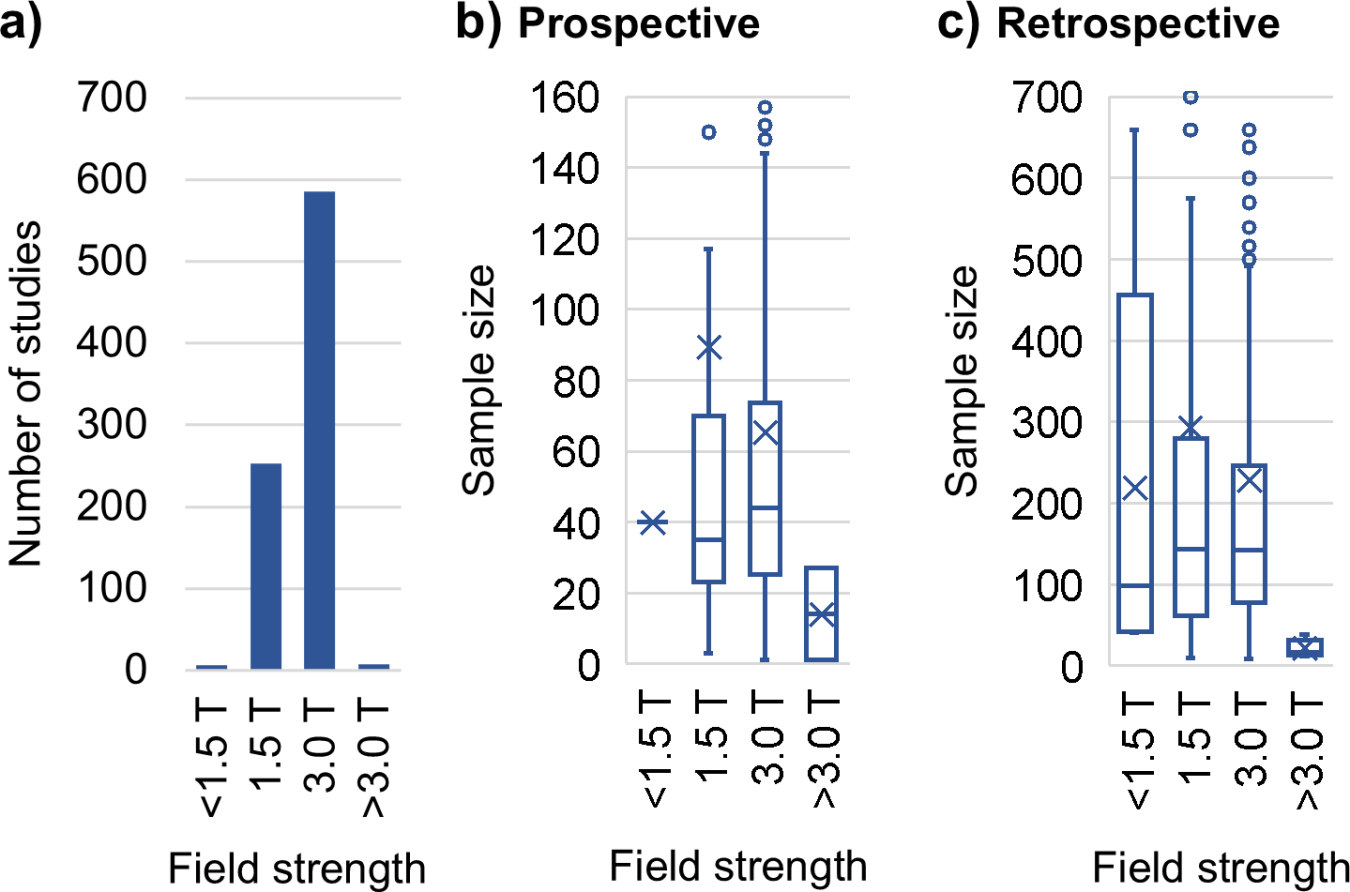
Field strength. Histogram of the number of studies per field strength (a) and matching boxplots of the sample sizes (displaying the minimum, maximum, first quartile, median, third quartile, mean, and outliers) in prospective (b) and retrospective (c) studies. The field strengths 0.2T, 0.55T, 5T, and 14.1T were summarized as < 1.5 T and > 3.0 T, respectively. Outliers are partially not visible.

Fig 5a illustrates the total number of studies using specific field strengths and S5 Table provides the respective numerical data. The most widely used field strengths were 3 T in 586 studies and 1.5 T in 253 studies. The field strengths of 0.2 T, 5 T, 14.1 T, and 0.55 T were used in one study each, while 7 T was used in five studies. Fige 5b depicts bar plots of the sample sizes in prospective studies for each field strength. Studies using a field strength of 1.5 T had a median sample size of 35 (mean = 89.3), and those using 3 T had a median sample size of 44 (mean =  65.3). The lowest median sample size, 14 (mean =  14, in two studies), was found in studies using field strengths >  3 T. Fig 5c shows the corresponding bar plot for the retrospective studies. For those using a field strength of 1.5 T, the median sample size was 143 (mean =  293), and for those with a field strength of 3 T, the median sample size was 142 (mean =  228.4). The studies with a field strength > 3.0 T had the lowest sample sizes (median =  17, mean =  21.2, in five studies).

### Contrast category

**Fig 6:**
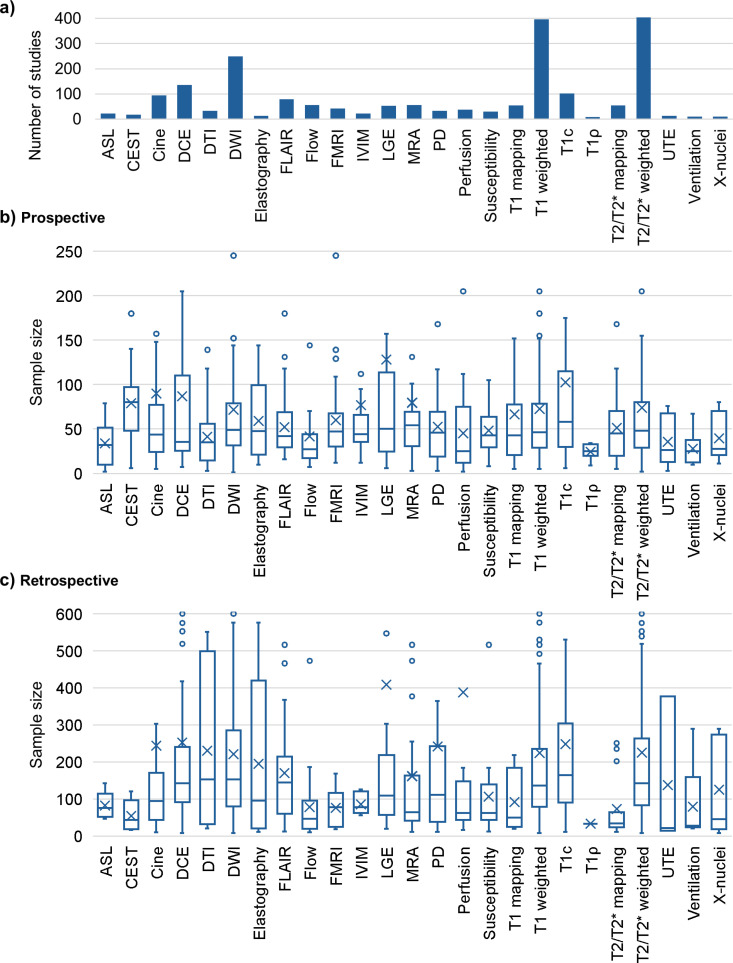
Contrast category. Histogram of the number of studies per contrast technique (a) and matching boxplots (displaying the minimum, maximum, first quartile, median, third quartile, mean, and outliers) of the sample sizes in prospective (b) and retrospective (c) studies. Outliers are partially not visible. Abbreviations: ASL =  arterial spin labeling; CEST =  chemical exchange saturation transfer; DCE =  dynamic contrast enhanced; DTI =  diffusion-tensor imaging; DWI =  diffusion-weighted imaging; FLAIR =  fluid-attenuated inversion recovery; FMRI =  functional magnetic resonance imaging; IVIM =  intravoxel incoherent motion imaging; LGE =  late gadolinium enhancement; MRA =  magnetic resonance angiogram; PD =  proton density; UTE =  ultrashort echo time.

The most widely used contrast categories were T2/T2 * weighted (404 studies, 55%) and *T1 weighted* (396 studies, 54%), followed by *DWI* (250 studies, 34.1%). The first two accounted for 39.3% of all contrast categories. Fig 6 illustrates the number of studies by contrast category and corresponding boxplots for the sample sizes in prospective and retrospective studies. S1 Table shows the respective numerical data. Most studies included multiple contrast techniques, with the mean number of techniques used per study being 2.8.

The highest median sample size for prospective studies was *CEST* (median =  80, mean =  78.9), followed by *T1c* (median =  58, mean =  102.4). The contrast categories with the lowest median sample sizes in prospective studies were *Ventilation* (median =  25, mean =  28), *Perfusion* (median =  25, mean =  45.2), and *T1ρ* (median =  25, mean =  24.4). The largest sample sizes for retrospective studies were also found in *T1c* (median =  164, mean =  247.8), followed by *DTI* (median =  153, mean =  230.8). The contrast category with the lowest median sample size in retrospective studies was *UTE,* with a median of 21 (mean =  137.3).

### Study type

**Fig 7:**
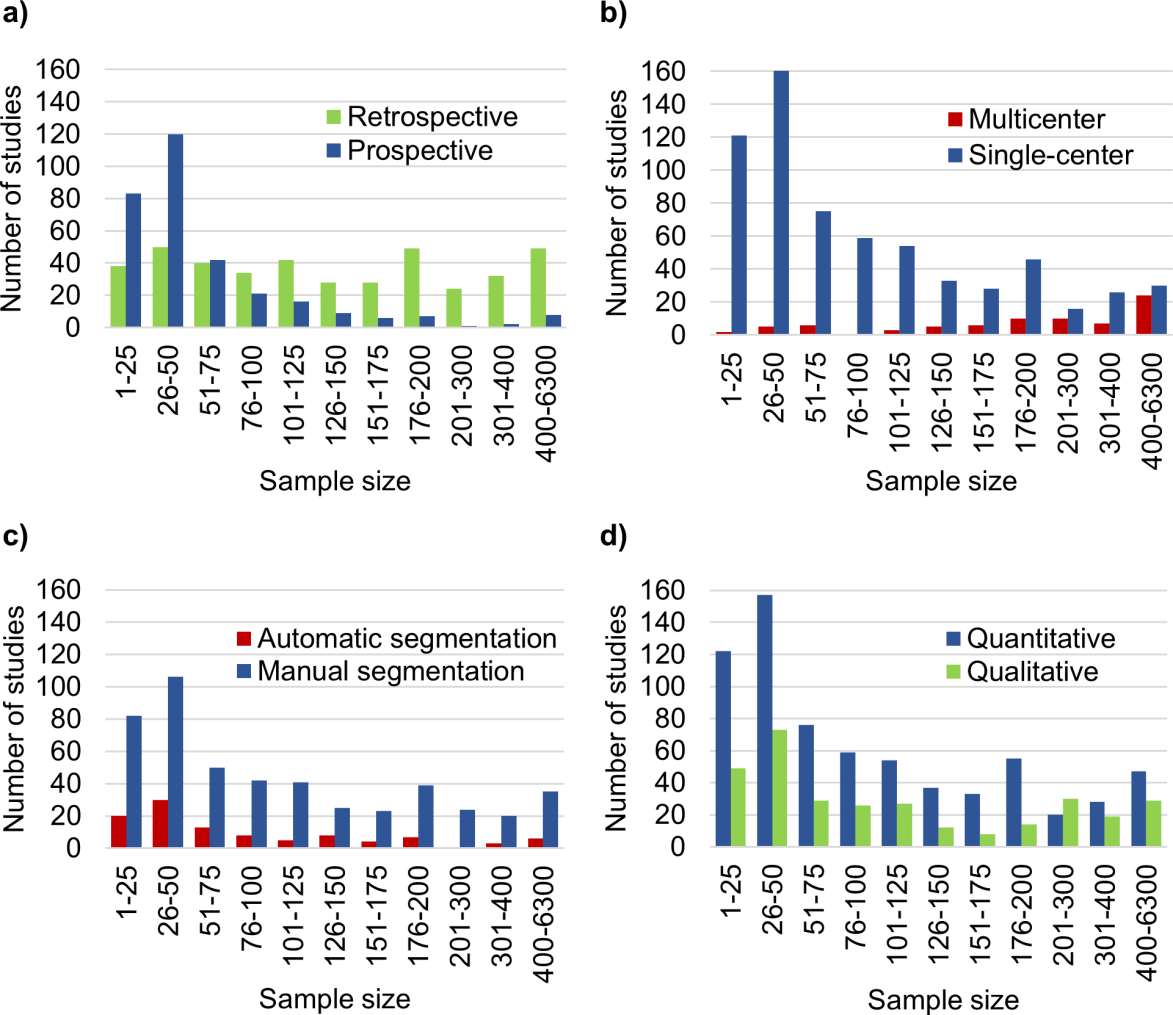
Sample sizes in different Categories. Histograms of the number of studies vs. sample sizes in prospective and retrospective studies (a). Histogram of the number of studies vs. sample sizes in multicenter and single-center studies (b). Histogram of the number of studies vs. sample sizes in studies using either automatic or manual segmentation for evaluation (c). Histogram of the number of studies vs. sample sizes in studies yielding either quantitative or qualitative data (d).

Fig 7a shows a histogram of the number of prospective (44.1%) and retrospective (56.4%) studies by sample size and S6 Table provides the respective numerical data. Some studies were divided into both a prospective and a retrospective part. Thus, the total number of retrospective and prospective studies exceeded the 734 included studies. The median sample size in retrospective studies was higher than in prospective ones, at 129 (mean =  228.8) vs. 41 (mean =  68.3). In bins with a sample size > 75, more studies were conducted retrospectively than prospectively. Retrospective studies had substantially larger maximum sample sizes (6229) [10] than prospective studies (1013 patients) [13]. Furthermore, 47.6% of the prospective studies also examined healthy volunteers, whereas only 22.0% of the retrospective studies did.

### Single vs. multicenter studies

Fig 7b shows the number of studies compared to sample sizes for multicenter and single-center studies. Only 10.6% of all studies were conducted using a multicenter approach. The median sample size was 240 (mean =  334.2) compared to 64 (mean =  136.7) in the single-center approach. Nevertheless, for large numbers of patients, especially in the range of 400–6300, the fraction of the multicenter studies compared to the single-center ones was increased (44.4% of 54 studies).

### Segmentation

Fig 7c shows the number of studies per sample size that used manual and automatic segmentations. A total of 488 studies (66.5%) used manual segmentations, compared to 104 (14.1%) that used automatic ones. The median sample size in studies using manual segmentation was 79 (mean =  170) and for automatic segmentation it was 55.5 (mean =  120).

### Quantitative vs. qualitative studies

Fig 7d depicts the number of studies compared to the sample sizes in studies that collected either quantitative or qualitative data. In 36.9% of the articles, both types of data were collected. Most studies (93.6%) yielded quantitative data, while 43.3% yielded qualitative data. The median sample size in quantitative studies was 70 (mean =  153.2), compared to 80 (mean =  192.2) in qualitative studies.

### Evaluation method

Table 1 lists the number of studies and the median (and mean) sample size per evaluation method category. *Manual segmentation* was the most commonly used method for evaluation (488 studies, 66.5%). *Categorization* was the second most common method with 192 studies (26.2%). The highest median sample size was found for *Machine learning* (median =  226, mean =  427.1) followed by *Radiomics* (median =  180, mean =  231.8). *Qualitative rating* had the lowest median sample size (median =  50, mean =  107.3). The median sample size for *Automatic* and *Semiautomatic segmentation* only differed by two (55.5 vs. 57.5 patients, respectively).

## Discussion

In this work, we assessed sample sizes and various study parameters in prospective and retrospective studies published in JMRI between 2020 and 2023.

Although the frequency of the sample size varied among the different categories (e.g., field strength and content category), each study represented an individual case and depended on study type, effect size, prevalence of the disease under investigation, and other parameters such as available scan time.

Retrospective studies had approximately a three-times higher median sample size (=129) than prospective ones (=41), which might be explained by the differences in the study designs and required statistical power. Prospective studies typically require larger resources and, therefore, smaller sample sizes may be sufficient to answer the research questions. In contrast, retrospective data (such as existing imaging data from a clinical repository) can facilitate analyzing a larger sample size in a less time-consuming manner. The retrospective study by Palau et al. [10] had the highest sample size with 6,229 patients. The authors performed 1.5 T vasodilator stress cardiac MRI on 2,370 female and 3,859 male patients with chronic coronary syndromes to evaluate the impact of sex on invasive coronary angiography (ICA). They found that ICA might be overused in men without ischemia. This is a compelling example of why a study with such a large sample size is needed when exploring small effect sizes in a given population. The study by Sarah Eskreis-Winkler et al. [14] had the second-highest sample size, with 5,224 patients. They aimed to develop a deep learning model for automated background parenchymal enhancement classification (in breast tissue) and used retrospective T1w contrast-enhanced and non-contrast enhanced breast MRI data measured at 1.5 T and 3 T. Supervised deep-learning algorithms often rely on large data sets (which is easier to obtain retrospectively) to train and evaluate neural networks [15]. In alignment with this, in our study, *Machine learning* had the highest median sample size among the evaluation methods (c.f. Table 1). *Radiomics*, a technique heavily reliant on sufficient data [16], had the second-highest median sample size among the investigated evaluation methods. Nevertheless, large sample sizes were the exception, as 90% of these studies used a sample size less than 350.

The largest sample size among the prospective studies was 1,013, published by Gavara et al. [13]. In that study, the authors used 1.5 T MRI to investigate the prognostic effect on the incidence of major adverse cardiac events. The large sample size was used to find group differences of an incidence value, which often requires a large data set. Notably, this study was also conducted using a multicenter approach, facilitating the recruitment of larger patient groups. However, this is an exceptionally large sample size, and most prospective studies (90%) used one below 151.

The retrospective study by Zhao et al. longitudinally investigated synthetic MRI parameters during neoadjuvant chemotherapy in breast cancer using the median sample size (=129) [17]. In that investigation, the authors extracted all patient data with invasive, ductal breast carcinomas during a time interval longer than two years and found that the synthetic T_1_ times change under treatment. Such retrospective studies frequently gather all available data within a specified time frame, with a consistent measurement and clinical protocol, to identify correlations between groups or, as in this case, observe time courses during treatment within a specific group. An example of a study using a median sample size close to the median sample size for prospective studies (=41) is the functional connectivity study by Hu et al., who investigated early-onset schizophrenia [18]. This study enrolled all patients (=48) within two years, matched them with 49 age- and gender-matched healthy volunteers, and showed multiple brain network abnormalities in the early-onset schizophrenia group.

A study with few included patients can also be well-designed. Various factors can justify such choices. For instance, some studies perform volunteer measurements to demonstrate the feasibility of their approach and added patient exams on top. One example is the study by Sneag et al. [19], in which the authors employed denoising methods on quantitative diffusion MRI to enhance the reliability of peripheral nerve diffusion measurements. This was tested in seven volunteers and three patients with peripheral neuropathy. Other studies with low sample sizes rely on rare patient data, e.g., imaging specific diseases in newborns. For example, Coll-Font et al.’s [20] study investigated kidney functioning with DCE MRI in newborns with hydronephrosis.

Interestingly, peaks in the sample sizes were observed at multiples of 10 in the investigated articles. This trend was also observed in technical MRI development studies in MRM [7], which might be interpreted as a cognitive bias towards round numbers [21].

When comparing content categories, the most common were *Neuro*, *Abdomen*, and *Cardiac*, emphasizing the importance of these anatomical regions in clinical MRI research and accounting for 52.2% of all studies (see Fig 3a). Neuroradiology is a large subspecialty within radiology and often organized in its own dedicated imaging facilities; thus, it constituted many of the studies (21.9%). The *Abdomen* category also comprised numerous studies, presumably because it contains a variety of anatomical organs. Furthermore, internal medicine, the corresponding clinical specialty, is the largest specialty (based on the number of active physicians in the United States, 2021) [22]. This is also true for the category *Cardiac,* as cardiovascular diseases account for a large proportion of overall mortality and disability and pose a substantial economic burden [23]. *Pediatrics* had the lowest prospective median sample size with 24 patients, possibly due to a smaller population to recruit from and greater challenges when imaging younger patients.

The predominant field strength used in the clinical MRI studies published in JMRI was 3 T, followed by 1.5 T, while the median sample size was similar for both field strengths. In contrast to method development studies [7], ultra-high field strengths, such as 7 T [24–29], were not commonly used in the investigated studies. Given the promising, specialized clinical applications of ultra-high field MRIs, this situation will likely change [30]. At the other end of the field strength spectrum [31, 32], it is possible that the use of low-field MRI scanners, such as 0.55 T, will increase due to greater access to MRI scans, reduced costs, and enhanced portability [33, 34].

In the contrast categories, it is notable that *T1 weighted* and T2/T2 * weighted were each represented in approximately 400 studies, accounting for 39.3% of all contrast categories (see Fig 6a). This may be partly explained by the fact that many studies, such as the APT-CEST study by Wamelink et al. [35] or the X-nuclei study by Mennecke et al. [36], used advanced techniques but also conventional sequences, such as T_1_-weighted imaging for registration and segmentation of different brain regions. The median number of patients varied slightly between the contrast categories, suggesting a link between smaller sample sizes when time-consuming or expensive techniques are used.

In total, 89.4% of the studies took a single-center approach, compared to 10.6% multicenter studies. However, the median sample size in multicenter studies (median =  240) was higher than in (median =  64) single-center ones. This indicates the possible need to take a multicenter approach to achieve a sufficient sample size and statistical power.

We found that manual segmentation was used more frequently than automatic segmentation as an evaluation method (488 vs. 104 studies, respectively) and involved a higher median sample size (79 vs. 55.5, respectively). This suggests that manual segmentation may be preferred (despite being labor-intensive) as automatic segmentation tools often require subsequent inspection or correction. The availability of automated tools may also play a role.

The higher number of quantitative studies (=687) than qualitative ones (=318) may be attributed to the fact that it is often easier to obtain quantitative data through automated processes while qualitative evaluations often require human interaction, such as rating, scoring, or categorization. In contrast, the median sample size of quantitative studies was slightly lower than in qualitative studies (70 vs. 80, respectively). Nevertheless, qualitative rating studies, which require a manual assessment, had the lowest median sample size (=50) among the investigated evaluation methods. This suggests that the complex and time-consuming rating process is more likely to be used in studies with fewer patients.

In a previous study published by our group [7], we investigated the number of healthy volunteers in technical development MRI studies. In contrast to the results of this study, the number of healthy volunteers was below 20 in 95.6% of all technical development MRI studies compared to the sample size in this study being below 75 in 50.5% of the studies. This suggests that clinically focused research, such as in *JMRI*, often relies on larger data sets as there is more variety in the appearance of diseased than healthy tissues. Moreover, technical development studies, as frequently published in *MRM*, often demonstrate the feasibility of a specific approach, whereas clinical MRI studies use novel techniques and apply them to patients or derive clinical data about a population. Compared to our previously published MRM study, here we investigated more study parameters, including the evaluation technique, whether a study used a multicenter or a single-center approach, and whether the study was retrospective or prospective. This also allowed us to investigate new trends, e.g., machine learning and its use of increased sample sizes.

## Limitations

There were some limitations in this study. First, in several categories, only a limited number of studies were available (e.g., field strengths > 3 T and < 1.5 T) and should be approached and interpreted cautiously as they do not permit reliable conclusions. Second, this study aimed to provide insights and an overview of current practices and methods in clinical MRI research. Hence, the results are not suitable for determining sample sizes in future studies and should only serve as an orientation after careful interpretation. For example, we did not consider important factors such as study design and disease prevalence, which can substantially change the statistics that should be used. For instance, finding a rare event in a screening study would require screening many individuals [37]. Third, we only performed a descriptive analysis of the extracted data, as the preconditions of the usually used tests (e.g., the Kruskal-Wallis test) assume that the data is not correlated (which is not met for contrast categories, where a single study may be classified into different contrast categories) [38]. Fourth, we evaluated the patient number only, not healthy subjects. This was not an issue in studies that compared two types of tissue alterations (for example, benign and malignant breast lesions [39]), but should be taken into account in studies that include a healthy control cohort (e.g., Pagé et al. who measured healthy controls in an MR elastography study on liver fibrosis [40]). Fifth, studies published in JMRI undergo rigorous peer review and, in our experience, are generally conducted to a high standard. However, we cannot guarantee that single studies may not draw statistically inappropriate conclusions. For example, some studies might have statistical shortcomings, such as not accounting for the effects of multiple comparisons, which are not always identified or corrected in the review process.

Finally, we chose to evaluate only the articles published in JMRI, as the focus was on current clinical MRI articles. Therefore, future studies should repeat and expand a similar analysis over a greater period to detect slowly progressing changes or expand the research to include additional MRI journals.

## Conclusion

There is wide variation in the sample sizes in the studies published by JMRI between 2020 and 2023, depending on study type, content category, and evaluation method. In clinical MRI studies, balancing statistical power and minimizing patient involvement is crucial and necessitates carefully choosing the sample size.

## Supporting information

S1 Table Excel Table providing the collected data, together with a Excel-based tool to extract specific parts of the data. (XLSX)

S2 Table Number of studies, percentage of studies and median, mean, minimal and maximal sample size per contrast category in retrospective (a) and prospective (b) studies. Abbreviations: ASL =  arterial spin labeling; CEST =  chemical exchange saturation transfer; DCE =  dynamic contrast enhanced; DTI =  diffusion-tensor imaging; DWI =  diffusion-weighted imaging; FLAIR =  fluid-attenuated inversion recovery; FMRI =  functional magnetic resonance imaging; IVIM =  intravoxel incoherent motion imaging; LGE =  late gadolinium enhancement; MRA =  magnetic resonance angiogram; PD =  proton density; UTE =  ultrashort echo time.
(DOCX)

S3 Table Number of articles published in JMRI from 2020 to 2023 which were included and excluded in this study (a). Number of studies and percentage of studies per binned sample size.
(DOCX)

S4 Table Number of studies, percentage of studies and median, mean, minimal and maximal sample size per content category in retrospective (a) and prospective (b) studies. (DOCX)

S5 Table Number of studies, percentage of studies and median, mean, minimal and maximal sample size per field strength in retrospective (a) and prospective (b) studies. (DOCX)

S6 Table Number of studies, percentage of studies and median, mean, minimal and maximal sample size in retrospective and prospective studies (a). Number of studies, percentage of studies and median, mean, minimal and maximal sample size in multicenter and single-center studies (b). Number of studies, percentage of studies and median, mean, minimal and maximal sample size in studies using either automatic or manual segmentation for evaluation (c). Number of studies, percentage of studies and median, mean, minimal and maximal sample size yielding either quantitative or qualitative data (d).
(DOCX)

## References

[1] van Beek EJR, Kuhl C, Anzai Y, Desmond P, Ehman RL, Gong Q, et al. Value of MRI in medicine: More than just another test?. J Magn Reson Imaging. 2019;49(7):e14–25. doi: 10.1002/jmri.26211 30145852 PMC7036752

[2] Djulbegovic B, Guyatt GH. Progress in evidence-based medicine: a quarter century on. Lancet. 2017;390(10092):415–23. doi: 10.1016/S0140-6736(16)31592-6 28215660

[3] Roletto A, Zanardo M, Bonfitto GR, Catania D, Sardanelli F, Zanoni S. The environmental impact of energy consumption and carbon emissions in radiology departments: a systematic review. Eur Radiol Exp. 2024;8(1):35. doi: 10.1186/s41747-024-00424-6 38418763 PMC10902235

[4] Parzonka K, Ndayishimiye C, Domagała A. Methods and Tools Used to Estimate the Shortages of Medical Staff in European Countries-Scoping Review. Int J Environ Res Public Health. 2023;20(4):2945. doi: 10.3390/ijerph20042945 36833641 PMC9957245

[5] Jones SR, Carley S, Harrison M. An introduction to power and sample size estimation. Emerg Med J. 2003;20(5):453–8. doi: 10.1136/emj.20.5.453 12954688 PMC1726174

[6] Eng J. Sample size estimation: how many individuals should be studied?. Radiology. 2003;227(2):309–13. doi: 10.1148/radiol.2272012051 12732691

[7] Hanspach J, Nagel AM, Hensel B, Uder M, Koros L, Laun FB. Sample size estimation: Current practice and considerations for original investigations in MRI technical development studies. Magn Reson Med. 2021;85(4):2109–16. doi: 10.1002/mrm.28550 33058265

[8] Valsasina P, Horsfield MA, Meani A, Gobbi C, Gallo A, Rocca MA, et al. Improved Assessment of Longitudinal Spinal Cord Atrophy in Multiple Sclerosis Using a Registration-Based Approach: Relevance for Clinical Studies. J Magn Reson Imaging. 2022;55(5):1559–68. doi: 10.1002/jmri.27937 34582062

[9] Imajo K, Kessoku T, Honda Y, Hasegawa S, Tomeno W, Ogawa Y, et al. MRI-Based Quantitative R2* Mapping at 3 Tesla Reflects Hepatic Iron Overload and Pathogenesis in Nonalcoholic Fatty Liver Disease Patients. J Magn Reson Imaging. 2022;55(1):111–25. doi: 10.1002/jmri.27810 34184822

[10] Palau P, Núñez J, Monmeneu JV, Lopez-Lereu MP, Gavara J, Rios-Navarro C, et al. Sex Effect in the Decision to Perform Invasive Coronary Angiography in Patients With Chronic Coronary Syndrome After Undergoing Vasodilator Stress MRI. J Magn Reson Imaging. 2022;56(6):1680–90. doi: 10.1002/jmri.28163 35344231

[11] Zhang Y, Yang C, Liang L, Shi Z, Zhu S, Chen C, et al. Preliminary Experience of 5.0 T Higher Field Abdominal Diffusion-Weighted MRI: Agreement of Apparent Diffusion Coefficient With 3.0 T Imaging. J Magn Reson Imaging. 2022;56(4):1009–17. doi: 10.1002/jmri.28097 35119776

[12] Liu Y, Wang S, Xu G, Zhou B, Zhang Y, Ye B, et al. Effectiveness and Accuracy of MRI-Ultrasound Fusion Targeted Biopsy Based on PI-RADS v2.1 Category in Transition/Peripheral Zone of the Prostate. J Magn Reson Imaging. 2023;58(3):709–17. doi: 10.1002/jmri.28614 36773016

[13] Gavara J, Marcos-Garces V, Lopez-Lereu MP, Monmeneu JV, Rios-Navarro C, de Dios E, et al. Magnetic Resonance Assessment of Left Ventricular Ejection Fraction at Any Time Post-Infarction for Prediction of Subsequent Events in a Large Multicenter STEMI Registry. J Magn Reson Imaging. 2022;56(2):476–87. doi: 10.1002/jmri.27789 34137478

[14] Eskreis-Winkler S, Sutton EJ, D’Alessio D, Gallagher K, Saphier N, Stember J, et al. Breast MRI Background Parenchymal Enhancement Categorization Using Deep Learning: Outperforming the Radiologist. J Magn Reson Imaging. 2022;56(4):1068–76. doi: 10.1002/jmri.28111 35167152 PMC9376189

[15] Alzubaidi L, Bai J, Al-Sabaawi A, Santamaría J, Albahri A, Al-dabbagh B. A survey on deep learning tools dealing with data scarcity: definitions, challenges, solutions, tips, and applications. Journal of Big Data. 2023;10(1):46.

[16] Gillies RJ, Kinahan PE, Hricak H. Radiomics: Images Are More than Pictures, They Are Data. Radiology. 2016;278(2):563–77. doi: 10.1148/radiol.2015151169 26579733 PMC4734157

[17] Zhao R, Du S, Gao S, Shi J, Zhang L. Time Course Changes of Synthetic Relaxation Time During Neoadjuvant Chemotherapy in Breast Cancer: The Optimal Parameter for Treatment Response Evaluation. J Magn Reson Imaging. 2023;58(4):1290–302. doi: 10.1002/jmri.28597 36621982

[18] Hu X, Wang S, Zhou H, Li N, Zhong C, Luo W, et al. Altered Functional Connectivity Strength in Distinct Brain Networks of Children With Early-Onset Schizophrenia. J Magn Reson Imaging. 2023;58(5):1617–23. doi: 10.1002/jmri.28682 36932678

[19] Sneag DB, Zochowski KC, Tan ET, Queler SC, Burge A, Endo Y, et al. Denoising of diffusion MRI improves peripheral nerve conspicuity and reproducibility. J Magn Reson Imaging. 2020;51(4):1128–37. doi: 10.1002/jmri.26965 31654542

[20] Coll-Font J, Afacan O, Chow JS, Lee RS, Stemmer A, Warfield SK, et al. Bulk motion-compensated DCE-MRI for functional imaging of kidneys in newborns. J Magn Reson Imaging. 2020;52(1):207–16. doi: 10.1002/jmri.27021 31837071 PMC7293568

[21] Coupland N. How frequent are numbers?. Language & Communication. 2011;31(1):27–37. doi: 10.1016/j.langcom.2010.09.001

[22] Executive Summary. 2023.

[23] Amini M, Zayeri F, Salehi M. Trend analysis of cardiovascular disease mortality, incidence, and mortality-to-incidence ratio: results from global burden of disease study 2017. BMC Public Health. 2021;21(1):401. doi: 10.1186/s12889-021-10429-0 33632204 PMC7905904

[24] Arts T, Siero J, Biessels G, Zwanenburg J. Automated assessment of cerebral arterial perforator function on 7T MRI. Journal of Magnetic Resonance Imaging. 2021;53(1):234–41. 32810376 10.1002/jmri.27304 PMC7754489

[25] van Tuijl RJ, Ruigrok YM, Geurts LJ, van der Schaaf IC, Biessels GJ, Rinkel GJE, et al. Does the Internal Carotid Artery Attenuate Blood-Flow Pulsatility in Small Vessel Disease? A 7 T 4D-Flow MRI Study. J Magn Reson Imaging. 2022;56(2):527–35. 34997655 10.1002/jmri.28062 PMC9546379

[26] Tang MCY, Jaarsma-Coes MG, Ferreira TA, Zwirs-Grech Fonk L, Marinkovic M, Luyten GPM, et al. A Comparison of 3 T and 7 T MRI for the Clinical Evaluation of Uveal Melanoma. J Magn Reson Imaging. 2022;55(5):1504–15. doi: 10.1002/jmri.27939 34652049 PMC9293452

[27] Arts T, Onkenhout LP, Amier RP, van der Geest R, van Harten T, Kappelle J, et al. Non-Invasive Assessment of Damping of Blood Flow Velocity Pulsatility in Cerebral Arteries With MRI. J Magn Reson Imaging. 2022;55(6):1785–94. doi: 10.1002/jmri.27989 34792263 PMC9298760

[28] Kamesh Iyer S, Moon BF, Josselyn N, Ruparel K, Roalf D, Song JW, et al. Data-Driven Quantitative Susceptibility Mapping Using Loss Adaptive Dipole Inversion (LADI). J Magn Reson Imaging. 2020;52(3):823–35. doi: 10.1002/jmri.27103 32128914 PMC8034229

[29] Wilson RL, Emery NC, Pierce DM, Neu CP. Spatial Gradients of Quantitative MRI as Biomarkers for Early Detection of Osteoarthritis: Data From Human Explants and the Osteoarthritis Initiative. J Magn Reson Imaging. 2023;58(1):189–97. doi: 10.1002/jmri.28471 36285338 PMC10126208

[30] Kraff O, Fischer A, Nagel AM, Mönninghoff C, Ladd ME. MRI at 7 Tesla and above: demonstrated and potential capabilities. J Magn Reson Imaging. 2015;41(1):13–33. doi: 10.1002/jmri.24573 24478137

[31] Perslev M, Pai A, Runhaar J, Igel C, Dam EB. Cross-Cohort Automatic Knee MRI Segmentation With Multi-Planar U-Nets. J Magn Reson Imaging. 2022;55(6):1650-63. 34918423 10.1002/jmri.27978 PMC9106804

[32] Campbell-Washburn A, Mancini C, Conrey A, Edwards L, Shanbhag S, Wood J, et al. Evaluation of hepatic iron overload using a contemporary 0.55 T MRI system. J Magn Reson Imaging. 2022;55(6):1855–63. 34668604 10.1002/jmri.27950 PMC9018883

[33] Arnold TC, Freeman CW, Litt B, Stein JM. Low-field MRI: Clinical promise and challenges. J Magn Reson Imaging. 2023;57(1):25–44. doi: 10.1002/jmri.28408 36120962 PMC9771987

[34] Heiss R, Nagel AM, Laun FB, Uder M, Bickelhaupt S. Low-Field Magnetic Resonance Imaging: A New Generation of Breakthrough Technology in Clinical Imaging. Invest Radiol. 2021;56(11):726–33. doi: 10.1097/RLI.0000000000000805 34132228

[35] Wamelink IJHG, Kuijer JPA, Padrela BE, Zhang Y, Barkhof F, Mutsaerts HJMM, et al. Reproducibility of 3 T APT-CEST in Healthy Volunteers and Patients With Brain Glioma. J Magn Reson Imaging. 2023;57(1):206–15. 35633282 10.1002/jmri.28239 PMC10084114

[36] Mennecke AB, Nagel AM, Huhn K, Linker RA, Schmidt M, Rothhammer V, et al. Longitudinal Sodium MRI of Multiple Sclerosis Lesions: Is there Added Value of Sodium Inversion Recovery MRI. J Magn Reson Imaging. 2022;55(1):140–51. doi: 10.1002/jmri.27832 34259373

[37] Bakker M, Lange Sv, Pijnappel R, Mann R, Peeters P, Monninkhof E. Supplemental MRI screening for women with extremely dense breast tissue. N Engl J Med. 2019;381(22):2091–102. 31774954 10.1056/NEJMoa1903986

[38] Bruce NG PD, Stanistreet DL. Probability Distributions, Hypothesis Testing, and Bayesian Methods. Quantitative Methods for Health Research; 2017. p. 477–527.

[39] Pistel M, Laun FB, Bickelhaupt S, Dada A, Weiland E, Niederdränk T, et al. Differentiating Benign and Malignant Breast Lesions in Diffusion Kurtosis MRI: Does the Averaging Procedure Matter?. J Magn Reson Imaging. 2022;56(5):1343–52. doi: 10.1002/jmri.28150 35289015

[40] Pagé G, Julea F, Paradis V, Vilgrain V, Valla D, Van Beers BE, et al. Comparative Analysis of a Locally Resampling MR Elastography Reconstruction Algorithm in Liver Fibrosis. J Magn Reson Imaging. 2023;58(2):403–14. doi: 10.1002/jmri.28543 36448664

