# Supplementary material for: Analysis of the sample size used in clinical MRI studies
Source: PLoS One. 2025 Mar 3;20(3):e0316611. doi: 10.1371/journal.pone.0316611 (PMC11875374; doi:10.1371/journal.pone.0316611)
Supplement: S2 Table — Abbreviations: ASL = arterial spin labeling; CEST = chemical exchange saturation transfer; DCE = dynamic contrast enhanced; DTI = diffusion-tensor imaging; DWI = diffusion-weighted imaging; FLAIR = fluid-attenuated inversion recovery; FMRI = functional magnetic resonance imaging; IVIM = intravoxel incoherent motion imaging; LGE = late gadolinium enhancement; MRA = magnetic resonance angiogram; PD = proton density; UTE = ultrashort echo time. (DOCX) [file pone.0316611.s002.docx]

**S2 Table**

1. **Retrospective**

| Contrasts | Number of studies | Percentage of studies | Sample size | | | |
| --- | --- | --- | --- | --- | --- | --- |
|  |  |  | Median | Mean | Min | Max |
| ASL | 6 | 0.8% | 75.5 | 82.8 | 46 | 142 |
| CEST | 5 | 0.7% | 43 | 54.4 | 16 | 121 |
| Cine | 45 | 6.1% | 94 | 243.6 | 10 | 6229 |
| DCE | 104 | 14.2% | 142.5 | 252.8 | 8 | 5224 |
| DTI | 8 | 1.1% | 153 | 230.8 | 20 | 551 |
| DWI | 154 | 21.0% | 152.5 | 221.4 | 8 | 1628 |
| Elastography | 5 | 0.7% | 95 | 195.2 | 11 | 576 |
| FLAIR | 43 | 5.9% | 145 | 169.7 | 12 | 755 |
| Flow | 25 | 3.4% | 46 | 77.8 | 10 | 473 |
| FMRI | 11 | 1.5% | 78 | 76 | 18 | 168 |
| IVIM | 7 | 1.0% | 78 | 85.4 | 56 | 126 |
| LGE | 23 | 3.1% | 109 | 408.8 | 19 | 6229 |
| MRA | 24 | 3.3% | 64.5 | 161.8 | 11 | 1157 |
| PD | 14 | 1.9% | 111 | 242.1 | 11 | 1073 |
| Perfusion | 20 | 2.7% | 62 | 388.1 | 16 | 6229 |
| Susceptibility | 16 | 2.2% | 62 | 106.56 | 12 | 516 |
| T1 mapping | 13 | 1.8% | 50 | 91.6 | 19 | 219 |
| T1 weighted | 235 | 32% | 136 | 223.7 | 8 | 5224 |
| T1c | 83 | 11.3% | 164 | 247.8 | 11 | 1225 |
| T1ρ | 1 | 0.1% | 33 | 33 | 33 | 33 |
| T2/T2* mapping | 15 | 2% | 34 | 72.9 | 11 | 251 |
| T2/T2* weighted | 259 | 35.2% | 142 | 225.2 | 8 | 2485 |
| UTE | 3 | 0.4% | 21 | 137.3 | 14 | 377 |
| Ventilation | 5 | 0.7% | 28 | 79 | 20 | 289 |
| X-nuclei | 5 | 0.7% | 45 | 125.6 | 8 | 289 |

1. **Prospective**

| Contrasts | Number of studies | Percentage of studies | Sample size | | | | |
| --- | --- | --- | --- | --- | --- | --- | --- |
|  |  |  | Median | Mean | | Min | Max |
| ASL | 17 | 2.3% | 32 | | 33.9 | 2 | 79 |
| CEST | 13 | 1.8% | 80 | | 78.9 | 6 | 180 |
| Cine | 50 | 6.8% | 43.5 | | 90.1 | 5 | 1013 |
| DCE | 32 | 4.4% | 35.5 | | 86.9 | 7 | 477 |
| DTI | 25 | 3.4% | 35 | | 41.4 | 3 | 139 |
| DWI | 96 | 13.1% | 49 | | 71.7 | 1 | 531 |
| Elastography | 8 | 1.1% | 47.5 | | 59 | 10 | 144 |
| FLAIR | 36 | 4.9% | 42 | | 52.1 | 16 | 180 |
| Flow | 32 | 4.4% | 27 | | 41.7 | 7 | 330 |
| FMRI | 32 | 4.4% | 47 | | 59.9 | 12 | 245 |
| IVIM | 16 | 2.2% | 44 | | 76.8 | 12 | 477 |
| LGE | 30 | 4.1% | 50 | | 128.2 | 6 | 1013 |
| MRA | 33 | 4.9% | 54 | | 79.36 | 3 | 567 |
| PD | 20 | 2.7% | 46 | | 52.5 | 3 | 168 |
| Perfusion | 19 | 2.6% | 25 | | 45.2 | 2 | 205 |
| Susceptibility | 14 | 1.9% | 43 | | 48.3 | 8 | 105 |
| T1 mapping | 41 | 5.6% | 43 | | 66.7 | 5 | 330 |
| T1 weighted | 162 | 22.1% | 46.5 | | 72.4 | 5 | 567 |
| T1c | 19 | 2.6% | 58 | | 102.4 | 6 | 402 |
| T1ρ | 7 | 1.0% | 25 | | 24.4 | 9 | 34 |
| T2/T2* mapping | 39 | 5.3% | 45 | | 51.4 | 5 | 168 |
| T2/T2* weighted | 147 | 20.0% | 48 | | 73.8 | 2 | 567 |
| UTE | 10 | 1.4% | 26.5 | | 35.6 | 3 | 76 |
| Ventilation | 6 | 0.8% | 25 | | 28 | 10 | 67 |
| X-nuclei | 6 | 0.8% | 27.5 | | 39.5 | 11 | 80 |
