# Supplementary material for: Analysis of the sample size used in clinical MRI studies
Source: PLoS One. 2025 Mar 3;20(3):e0316611. doi: 10.1371/journal.pone.0316611 (PMC11875374; doi:10.1371/journal.pone.0316611)
Supplement: S3 Table — Number of studies and percentage of studies per binned sample size. (DOCX) [file pone.0316611.s003.docx]

**S3 Table**

**a)**

| Year | Included | Excluded | All |
| --- | --- | --- | --- |
| 2020 | 169 | 89 | 258 |
| 2021 | 194 | 87 | 281 |
| 2022 | 172 | 74 | 246 |
| 2023 | 199 | 64 | 263 |
| ∑ | 734 | 314 | 1048 |

**b)**

| Number of patients | Number of studies | Percentage of studies |
| --- | --- | --- |
| 1-25 | 123 | 16.8% |
| 26-50 | 172 | 23.4% |
| 51-75 | 77 | 11.4% |
| 76-100 | 62 | 8.4% |
| 101-125 | 59 | 8.0% |
| 126-150 | 38 | 5.2% |
| 151-175 | 34 | 4.6% |
| 176-200 | 21 | 2.9% |
| 201-225 | 21 | 2.9% |
| 226-250 | 19 | 2.6% |
| 251-275 | 10 | 1.4% |
| 276-300 | 10 | 1.4% |
| 301-325 | 8 | 1.1% |
| 326-350 | 7 | 1.0% |
| 351-375 | 8 | 1.1% |
| 376-400 | 10 | 1.4% |
| 401-425 | 4 | 0.5% |
| 426-450 | 1 | 0.1% |
| 451-475 | 4 | 0.5% |
| 476-500 | 4 | 0.5% |
| 501-525 | 4 | 0.5% |
| 526-550 | 6 | 0.8% |
| 551-575 | 6 | 0.8% |
| 576-600 | 2 | 0.3% |
| 601-625 | 0 | 0.0% |
| 626-650 | 1 | 0.1% |
| 651-675 | 1 | 0.1% |
| 676-700 | 1 | 0.1% |
| 701-800 | 7 | 1.0% |
| 801-900 | 1 | 0.1% |
| 901-1000 | 1 | 0.1% |
| >1000 | 12 | 1.6% |
| All | 734 | 100% |
