# Supplementary material for: Analysis of the sample size used in clinical MRI studies
Source: PLoS One. 2025 Mar 3;20(3):e0316611. doi: 10.1371/journal.pone.0316611 (PMC11875374; doi:10.1371/journal.pone.0316611)
Supplement: S4 Table — (DOCX) [file pone.0316611.s004.docx]

**S4 Table**

1. **Retrospective**

| Content category | Number of studies | Percentage of studies | Sample size | | | |
| --- | --- | --- | --- | --- | --- | --- |
|  |  |  | Median | Mean | Min | Max |
| Abdomen | 77 | 10.5% | 180 | 206.1 | 11 | 1069 |
| Breast | 42 | 5.7% | 157 | 373.4 | 10 | 5224 |
| Cardiac | 49 | 6.7% | 109 | 273.1 | 10 | 6229 |
| Chest | 4 | 0.5% | 50 | 61 | 21 | 123 |
| Head and Neck | 30 | 4.1% | 159.5 | 362.2 | 44 | 2485 |
| Interventional | 1 | 0.1% | 309 | 309 | 309 | 309 |
| Musculoskeletal | 35 | 4.8% | 103 | 206.9 | 14 | 1073 |
| Neuro | 80 | 10.9% | 98.5 | 155.3 | 12 | 755 |
| Pediatrics | 12 | 1.6% | 108 | 123.6 | 20 | 492 |
| Pelvis | 54 | 7.4% | 155.5 | 253.0 | 8 | 1628 |
| Safety | 1 | 0.1% | 125 | 125 | 125 | 125 |
| Technical | 10 | 1.4% | 57 | 169.2 | 12 | 553 |
| Thoracic | 8 | 1.0% | 29 | 89.4 | 19 | 289 |
| Vascular | 10 | 1.4% | 52.5 | 96.8 | 11 | 473 |
| Whole Body | 1 | 0.1% | 66 | 66 | 66 | 66 |

1. **Prospective**

| Content category | Number of studies | Percentage of studies | Sample size | | | |
| --- | --- | --- | --- | --- | --- | --- |
|  |  |  | Median | Mean | Min | Max |
| Abdomen | 39 | 5.3% | 48 | 64.8 | 1 | 397 |
| Breast | 15 | 2.0% | 60 | 102.3 | 5 | 477 |
| Cardiac | 58 | 7.9% | 41.5 | 87.5 | 5 | 1013 |
| Chest | 13 | 1.7% | 46 | 66.2 | 10 | 245 |
| Head and Neck | 17 | 2.3% | 41 | 58.6 | 10 | 293 |
| Interventional | 1 | 0.1% | 30 | 30 | 30 | 30 |
| Musculoskeletal | 27 | 3.6% | 35 | 49.7 | 2 | 210 |
| Neuro | 82 | 11.1% | 40 | 56.8 | 6 | 551 |
| Pediatrics | 10 | 1.4% | 24 | 50.1 | 11 | 154 |
| Pelvis | 19 | 2.5% | 66 | 105.2 | 5 | 531 |
| Safety | 1 | 0.1% | 40 | 40 | 40 | 40 |
| Technical | 8 | 1.0% | 44.5 | 52.6 | 6 | 175 |
| Thoracic | 6 | 0.8% | 27 | 32.7 | 13 | 78 |
| Vascular | 25 | 3.4% | 32 | 72.9 | 3 | 567 |
| Whole Body | 2 | 0.2% | 32 | 32 | 11 | 53 |
