# Supplementary material for: Analysis of the sample size used in clinical MRI studies
Source: PLoS One. 2025 Mar 3;20(3):e0316611. doi: 10.1371/journal.pone.0316611 (PMC11875374; doi:10.1371/journal.pone.0316611)
Supplement: S5 Table — (DOCX) [file pone.0316611.s005.docx]

**S5 Table**

1. **Retrospective**

| Field strength | Number of studies | Percentage of studies | Sample size | | | |
| --- | --- | --- | --- | --- | --- | --- |
|  |  |  | Median | Mean | Min | Max |
| <1.5 T | 5 | 0.7% | 98 | 218.8 | 41 | 659 |
| 1.5 T | 177 | 24.1% | 143 | 293 | 10 | 6229 |
| 3.0 T | 332 | 45.2% | 142 | 228.4 | 8 | 5224 |
| >3.0 T | 5 | 0.7% | 17 | 21.2 | 12 | 38 |

| Field strength | Number of studies | Percentage of studies | Sample size | | | |
| --- | --- | --- | --- | --- | --- | --- |
|  |  |  | Median | Mean | Min | Max |
| <1.5 T | 1 | 0.1% | 40 | 40 | 40 | 40 |
| 1.5 T | 79 | 10.7% | 35 | 89.3 | 3 | 1013 |
| 3.0 T | 256 | 34.8% | 44 | 65.3 | 1 | 567 |
| >3.0 T | 2 | 0.2% | 14 | 14 | 1 | 27 |

1. **Prospective**
