# Supplementary material for: Analysis of the sample size used in clinical MRI studies
Source: PLoS One. 2025 Mar 3;20(3):e0316611. doi: 10.1371/journal.pone.0316611 (PMC11875374; doi:10.1371/journal.pone.0316611)
Supplement: S6 Table — Number of studies, percentage of studies and median, mean, minimal and maximal sample size in multicenter and single-center studies (b). Number of studies, percentage of studies and median, mean, minimal and maximal sample size in studies using either automatic or manual segmentation for evaluation (c). Number of studies, percentage of studies and median, mean, minimal and maximal sample size yielding either quantitative or qualitative data (d). (DOCX) [file pone.0316611.s006.docx]

**S6 Table**

**a)**

| Study Type | Number of studies | Percentage of studies | Sample size | | | |
| --- | --- | --- | --- | --- | --- | --- |
|  |  |  | Median | Mean | Min | Max |
| Retrospective | 414 | 56.4% | 129 | 228.8 | 8 | 6229 |
| Prospective | 323 | 44.0% | 41 | 68.3 | 1 | 1013 |

| Center | Number of studies | Percantage of studies | Sample size | | | |
| --- | --- | --- | --- | --- | --- | --- |
|  |  |  | Median | Mean | Min | Max |
| Multicenter | 78 | 10.6% | 240 | 334.2 | 24 | 1399 |
| Single-center | 656 | 89.4% | 64 | 136.7 | 1 | 6229 |

**b)**

| Evaluation method | Number of studies | Percantage of studies | Sample size | | | |
| --- | --- | --- | --- | --- | --- | --- |
|  |  |  | Median | Mean | Min | Max |
| Automatic segmentation | 104 | 14.1% | 55.5 | 120 | 8 | 1628 |
| Manual segmentation | 488 | 66.5% | 79 | 170 | 1 | 6229 |

**c)**

| Category | Number of studies | Percantage of studies | Sample size | | | |
| --- | --- | --- | --- | --- | --- | --- |
|  |  |  | Median | Mean | Min | Max |
| Quantitative | 687 | 93.6% | 70 | 153.2 | 1 | 6229 |
| Qualitative | 318 | 43.3% | 80 | 192.2 | 1 | 6229 |

**d)**
